# Functional Constructivism Approach to Multilevel Nature of Bio-Behavioral Diversity

**DOI:** 10.3389/fpsyt.2021.641286

**Published:** 2021-10-27

**Authors:** Irina Trofimova

**Affiliations:** Laboratory of Collective Intelligence, Department of Psychiatry and Behavioural Neurosciences, McMaster University, Hamilton, ON, Canada

**Keywords:** neurotransmitters, temperament, psychopathology, functional ensemble of temperament model, consistent behavioral patterns, taxonomies, diagonal evolution

## Abstract

Attempts to revise the existing classifications of psychiatric disorders (DSM and ICD) continue and highlight a crucial need for the identification of biomarkers underlying symptoms of psychopathology. The present review highlights the benefits of using a Functional Constructivism approach in the analysis of the functionality of the main neurotransmitters. This approach explores the idea that behavior is neither reactive nor pro-active, but constructive and generative, being a transient selection of multiple degrees of freedom in perception and actions. This review briefly describes main consensus points in neuroscience related to the functionality of eight neurochemical ensembles, summarized as a part of the neurochemical model Functional Ensemble of Temperament (FET). None of the FET components is represented by a single neurotransmitter; all neurochemical teams have specific functionality in selection of behavioral degrees of freedom and stages of action construction. The review demonstrates the possibility of unifying taxonomies of temperament and classifications of psychiatric disorders and presenting these taxonomies formally and systematically. The paper also highlights the multi-level nature of regulation of consistent bio-behavioral individual differences, in line with the concepts of diagonal evolution (proposed earlier) and Specialized Extended Phenotype.

## The Complexity of Neurochemical Markers of Bio-Behavioral Diversity Requires Formalisms of Complexity Science

### Challenging Complexity of Neurochemical Biomarkers for Bio-Behavioral Traits

Attempts to revise the existing classifications of psychiatric disorders (DSM and ICD) continue and highlight a crucial need for the identification of biomarkers underlying symptoms of psychopathology. Studies of neurochemical biomarkers are often overshadowed by studies in neuroimaging and often require very invasive methods. Yet most psychiatric interventions use neurochemical, not neuro-anatomic, methods. The preference for biomarkers of psychopathology oriented on brain structures is associated with another common weakness of models in computational psychiatry and psychology: their static presentation of the systems of behavioral regulation. This presentation assumes a permanent functionality of these structures by analogy of functionality of mechanical parts of a car. As another approach to taxonomies, too much trust is also given to the ability of linear statistical methods to derive bio-behavioral classifications (1). Such approaches often underestimate the generative, constructive nature of neurophysiological and psychological processes and the challenges that their transience poses for classification purposes (1–3).

The aim of this review was to demonstrate the benefits of the constructivism approach to taxonomies of bio-behavioral individual differences that are based on neurochemical systems.

The need to identify neurochemical biomarkers of bio-behavioral individual differences has been appreciated for a long time. It has been over 2,500 years since Hippocrates and then Galen suggested that ratios within the interaction between vital bodily fluids can be the basis of temperament types. The extreme deviations of these types subsequently resembled psychiatric disorders (depression, ADHD, mania, social withdrawal). The practice of (neuro) chemical treatment of these deviations proved the validity of this theory in general: shifting neurochemical cycles of neurotransmitter activities indeed leads to changes in behavioral patterns.

However, the “devil is in the details,” and the task of sorting out the functionality of neurotransmitters appeared to be far from trivial. More than a dozen of non-peptide and over 100 peptide neurotransmitters have been identified, each having different functionality and distribution in the nervous system. In addition, many of these neurotransmitters have a diversity of receptors, each having different functionality and location pattern. For example, there are (so far discovered) 5 types of dopamine (DA) receptor, 9 types of adrenergic receptor, 14+ types of serotonin (5-HT) receptor, two families of acetylcholine (ACh) receptor (each having 5–12 subtypes). A similar diversity of receptors has been found for histamine, Gamma-Amino-Butyric Acid (GABA), glutamate (Glu) and the endogenous opioids (4–6). Moreover, neurotransmitters regulate one another's activity in a contingent manner via several mechanisms with different release patterns and different mediators depending on the intensity of stimulation and the location and density of receptors. The same two NTs can be rivals under one condition (or location), suppressing each other's release, or partners under another condition/location, having a co-release. Recent development in neuroscience also showed that small non-coding RNAs (in particular microRNAs) also play a significant role in the translational regulation at the synapses and so can be potential biomarkers of individual differences in behavior, especially in psychopathology (7). Finally, neurotransmitters often use so-called “volume transmission,” i.e., transmission in which their NT is released to the extracellular space and acts on whatever neurons are there receptive to it (4, 8–11).

In other words, there is a seemingly disorganized “soup” of neurochemical biomarkers of bio-behavioral traits, and even the list of these traits remains a topic of hot debates (1, 12). The use of lengthy texts descriptions of this complexity seems to be not a very efficient way to handle it, and, therefore, there is a need for more systematic approaches using formalisms and language of Complexity science, non-linear dynamics and synergetics (13–16). The author's own search for mathematical formalisms of this complexity using existing models of non-linear dynamics and complex systems involved multiple approaches, including factor analysis (17, 18), multi-agent modeling of stochastic clustering (19–21), formal presentation of diversity (21) and analysis of functional differentiation (22, 23). These approaches had some use, but overall current mathematics appeared to be insufficient for the formal description of natural systems of behavioral regulation. As in any natural system, the main challenge was transience, observed in the absence of permanent connectivity, single-time emergence of states, multiplicity of feedback, and contingency mechanisms.

The goal of this review is to highlight the correspondence of functionality of neurochemical systems with the universal functional aspects of the common tasks that are shaped by bio-social environment. Aspects of this functionality have been presented earlier as the neurochemical model *Functional Ensemble of Temperament* (FET) (24–28). This review highlights only few, primarily global aspects of behavioral regulation, omitting a big number of more detailed functional relationships within these neurochemical systems.

In medical sciences, it is mandatory to learn how regulatory systems operate and interact *in a healthy state* in order to classify pathology related to these systems. The same approach is taken here. The bulk of this paper analyses functionality of neurochemical biomarkers in general, in relatively healthy cases, followed by a brief discussion of a possible classification of psychopathology.

The functionality of neurochemical systems is labeled here with formal symbols to facilitate a more compact mathematical analysis in the future in the field of computational psychiatry. The text, admittedly, looks strange with these symbols but the complexity of neurochemical biomarkers and the wealth of findings in neuroscience call for compact formal languages, to facilitate scientific discussions on this matter. Here we also use the abbreviation CBP for consistent bio-behavioral patterns (i.e., temperament traits and symptoms of psychopathology) and the expression behavioral alternatives, or behavioral elements that include not only motor actions but also products of perception, images, thoughts, decisions, recalled elements, dispositions, programs of actions, etc. (i.e., all products of the activity of nervous system that are necessary to construct a behavioral act).

### Functional Constructivism

The overall approach that the FET is based on is called *Functional Constructivism*.

*Constructivism* approach in psychology began in the 1930s (29–32) and explores the idea that behavior is neither reactive nor pro-active, but instead constructive and generative, similar to writing a signature every time differently. All processes and actions are generated as the unique integration of behavioral alternatives selected out of current multiple degrees of freedom and sequenced for their relevance. According to the dictionary of the American Psychological Association, *behavioral integration* refers to “the combination of separate individual behaviors into a synchronized or coordinated behavioral unit.” The generative, constructive nature of behavioral regulation and the benefits of the constructivism approach were noticed at many levels within different bio-behavioral sciences, including neurophysiology (33–40), neurochemistry (41, 42), developmental and educational psychology (43–46), ecological psychology (47), psychological modeling (13, 14, 16, 48–50), psychology of cognition (15, 32, 36, 51–53), and psychology of emotions (54–58).

As a part of the constructivism approach, “*Functional Constructivism*” (FC) focuses on the functional differentiation of the universal features of behavioral construction and applies it to the classification (partitioning) of neurobehavioral regulatory systems. Currently, there are multiple opinions on how neurochemical regulatory systems should be partitioned and named (e.g., “limbic system,” “attentional networks,” “sensory-motor networks,” default network,” “reward network,” etc). The FET model offers one more partitioning based on FC and also evolutionary theory. If behavior is presented here as a constructive, generative process, then it is natural to see the correspondence between principles of behavioral regulation and general principles of evolution, such as:

1. Natural selection, transience, and neutrality of emerging configurations (23).
2. The multi-level nature of selection, co-evolution, and convergent evolution principles, when environment and species regulate each other's evolution (23).
3. Structuring of “selectors” into reproducible units with limited d.f.—such as genes or memes, that we previously called “cruise controls” (23, 59).

Before detailing those neurochemical systems and their deviations in psychiatric disorders that are relevant for psychiatrists, let's briefly cover the second principle of multi-level selection since this volume is specialized in bio-social complexity, and highlight the benefits of the concept of *Specialized Extended Phenotype*.

## Biosocial Complexity: Multi-Level Selection, Specialized Extended Phenotype and Dievolution in Bio-Behavioral Regulation

Bio-social complexity of behavioral regulation results from the interaction between two types of selectors: bio-chemical and socio-cultural. The diversity of socio-cultural environments create differences in advantages and disadvantages for people's degrees of freedom in behavior. In discussing the improvement of classifications of bio-behavioral individual differences, the relevant questions to ask are: with all socio-cultural regulators equal (for example, in siblings from the same family) why do people respond to these “selectors and regulators” differently? Why are some of them more susceptible to social norms than others? Why do they use different activities to develop their skill sets? And more intriguing question is: do people's biochemical individual differences, in turn, influence the setups of their functional environments? Here are several important points in this regard.

**Figure 1:**
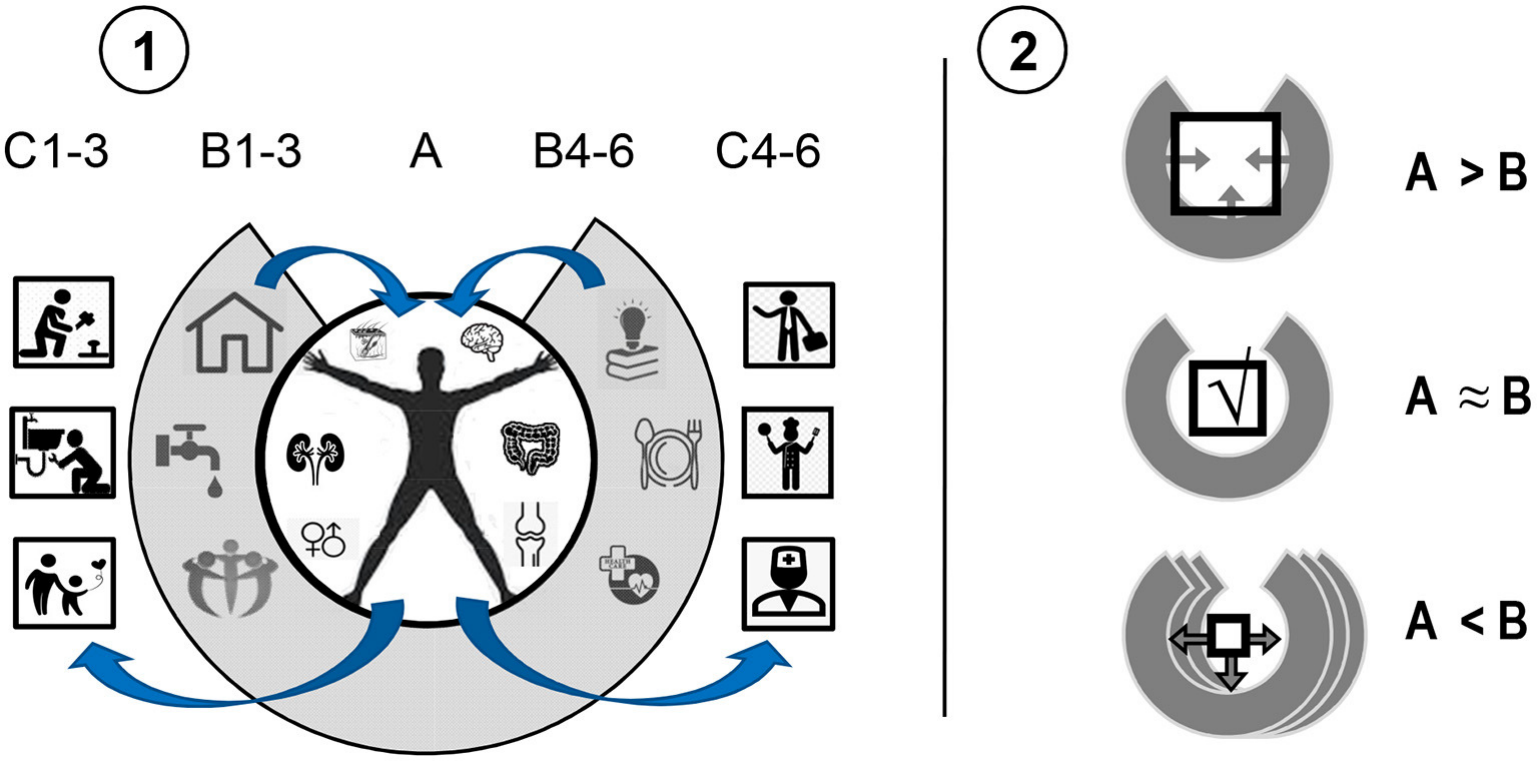
The concept of interactive Extended Phenotype. **(1)**: (A) in order to survive, humans receive resources from (B) the infrastructure provided by a society to respond to their vital needs. This makes the environmental resource-infrastructure an extended part of an individual's body. In return, the infrastructure uses individuals for producing actions, services and objects supporting this infrastructure (C). **(2)**: Excessive abilities of an individual are trimmed by societal infrastructure or force the infrastructure to expand its expectations (A > B). In cases of disabilities (A < B), additional environmental resources are required, often leading to the development of environmental infrastructure.

**Figure 2:**
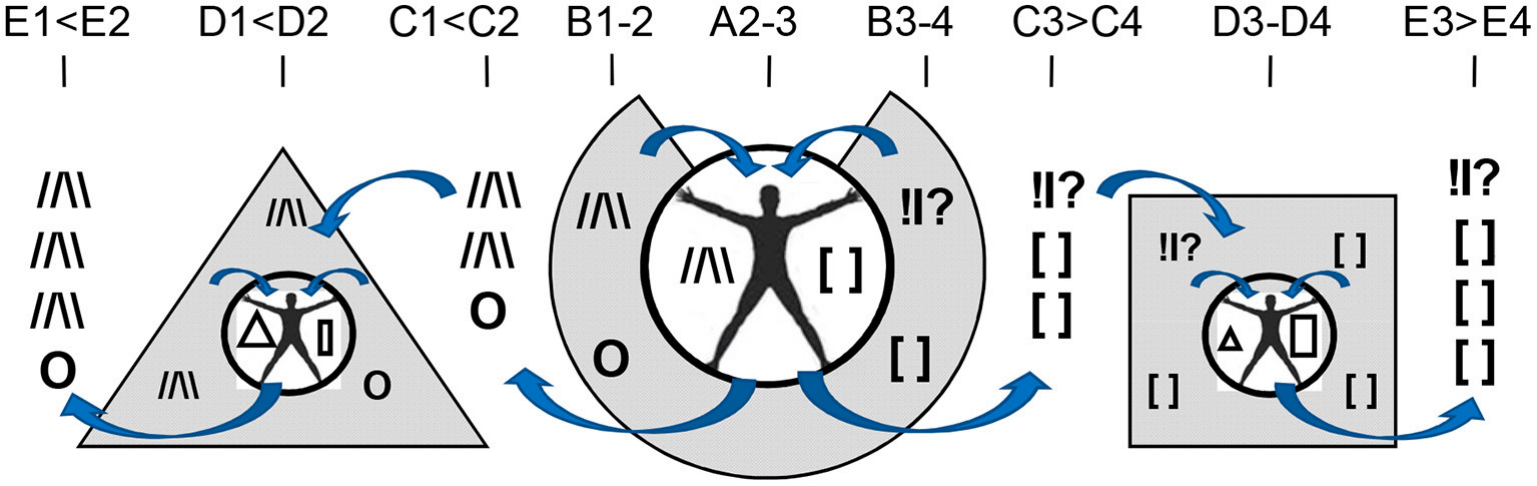
An illustration of the concept of *Specialized Extended Phenotype (SEP*). People with two types of bio-behavioral (dis)abilities **(A)** interact with the environment **(B)** requiring four types of tasks and corresponding abilities. Individuals produce actions **(C)** in a biased way, primarily in line with their specific capacities, and, therefore, not following all the requirements. This, in turn, contributes to *SEPs* as the more specialized parts of the environmental infrastructure **(D)** providing support for specific (dis)abilities. SEP-compatible actions of an individual **(C,E)**, therefore, contribute to the existence and development of specialized “industries” in the environmental infrastructure **(D)**, which, in turn, reinforce more actions of this type. Geometric shapes refer to both, socially desirable functional actions and to dysfunctional actions (e.g., seen in addiction) that are entangled with SEPs supporting these actions. In this example, two types of SEPs are developed: supporting orientation to novelty (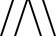) and plasticity of actions ([]). Numbers formally differentiate between four types of (dis)abilities.

1. With all things equal, the preferences in behavior are given to those tasks and regulators that are compatible with the neurobiological capacities. In other words, people chose actions that they can do, not what they should do or planned to do.
2. After the program of action has been selected, and executed, the resulting actions leave a trace (experience) in several parts of the nervous system. These learned units of behavior become new capacities, affecting future choices in behavior, setting up the epigenetic path in the development of consistent behavioral patterns (CBPs).
3. Actions do not happen in a vacuum. The most commonly discussed aspect of bio-social regulation is socio-cultural regulation. Indeed, survival of a human body largely depends on supplies of water, food, shelter, medical care, peers, knowledge, etc. (i.e., vital resources that a human society's infrastructure provides). In order to obtain these resources, humans have to adapt their actions to the shape of objects and rules of behavior, learning specific behavioral programs of actions from birth. The basic service infrastructure, forced everybody to learn the same behavioral package, in exchange for access to society's resources. The use of this supply-infrastructure provides a de facto extension of the human body as an instrument of behavioral construction. The dependency of individual behavior on it and the resulting pro-societal evolution of the human psyche are highlighted in the theory of Extended Phenotype offered by Richard Dawkins (60). An important aspect of the Extended Phenotype principle is that in most cases, people's actions feedback to the physical, social and economic infrastructure in which they produced these actions (Figure 1-1). People's habitual behavior and skills become the units of “production” supporting the functional cycles of the economy and society. Since people's capacities are limited, the expectations and regulations of social infrastructure are also limited. In an ideal world, for example, everyone works hard, supports one another and plays by the rules; in reality, however, there is a wide spectrum of disabilities and antisocial behavior that requires the societal infrastructure to have social services, law enforcement and assisted living arrangements (Figure 1-2). Therefore, there are back and forth adjustments between requests from the social infrastructure for particular behaviors and consideration by that infrastructure of people's behavioral capacities, which shapes its expectations.
4. The two-way adjustments between features of neurobiological capacities and the environmental infrastructure can generate segregated functional cycles, “functional bubbles,” consisting of specialized parts of this infrastructure. The theory of Extended Phenotype can be then expanded to the *Specialized Extended Phenotype (SEP)* theory, to acknowledge the reinforcement of specialized sub-infrastructures of society by bio-behavioral capacities (Figure 2). Individual differences in these capacities make people prefer specific types of activities (for example, manual work, verbal, or mental activities) as it is easier for them to perform them. Society develops specialized social and economic infrastructures for these different types of activities, and so people with different abilities eventually settle with those specialized parts of the Extended Phenotype that are most compatible, creating socio-functional cycles. Various groups in society exercise different standards, work with specific objects and services and promote the infrastructure that corresponds to their capacities. Biases in the reinforcement of specific types of neuronal capacities in different professionals promotes SEP segregation. Professional differentiation and special services, therefore, might be arising from the diversity of bio-behavioral capacities of individuals that compose given societies. Despite of the equal access to various activities for all people in civilized societies, people use only a small portion of this access, mostly compatible with their bio-behavioral abilities, living in “bubbles” formed by their professional environments and support networks. These SEP bubbles reinforce the CBPs that include behavioral orientation to specific objects, settings, partners, friends, groups in society as their regulators, compatible with people's capacities and needs.
5. The levels of behavioral regulation are often presented vertically, with the neurochemical systems and cells at the bottom, and with societies and economies at the top. There are also phenomena of “horizontal” regulation described in the literature on Complex Systems, in phenomena such as collective intelligence effects (61), mass action (15, 16, 19–21), holographic effects (38), predator-prey and host-parasite interactions (13, 14), and functional differentiation (22, 23). These phenomena illustrate the regulation of performance of systems not by regulators positioned above or below but by regulators at the same level of complexity—peers, predators, family members, etc. The reason we mention these vertical and horizontal factors for the selection of behavior is to show the benefits of the “*diagonal evolution*” concept (*dievolution*) (23, 59), applicable not only to the evolution of the human psyche but also to the generation of behavior.

The main idea of dievolution is that evolution (and construction of behavior) does not proceed as Lego-block compositions of smaller- to larger blocks of organization (for example, from chemicals to cells, from the unification of cells to multi-cellular bodies). Instead, it proceeds as the coevolution of multiple levels of organization driven by their compatibility. Consistent compatibility across multiple levels of regulation improves the sustainability of specific arrangements as the result of multiple iterations between levels. As an example of the evolution of neurobiological capacities, complex manual work developed the human striatum but also an infrastructure of instruments, trades, schooling, etc associated with this work. Similarly, differences among species in the distribution of NT releasing sites and receptors could be explained by differences in their behavioral tasks (4, 62). Verbal activities developed the human temporal lobes and adaptability to uncertainty along with the infrastructure supporting verbal exchanges (books, libraries, languages, etc). A diversity of environments developed the frontal lobes and its infrastructure of science. Several temperament researchers have suggested an activity-specific differentiation of traits regulating physical, verbal-social, and mental aspects of tasks (63–67).

## Natural Selection in Behavior: From Orientation to Integration, and Their Neurochemical Biomarkers

### Neurochemical Biomarkers of Behavioral Orientation, as 1st Stage of Selection of Degrees of Freedom in Behavior

**Figure 3:**
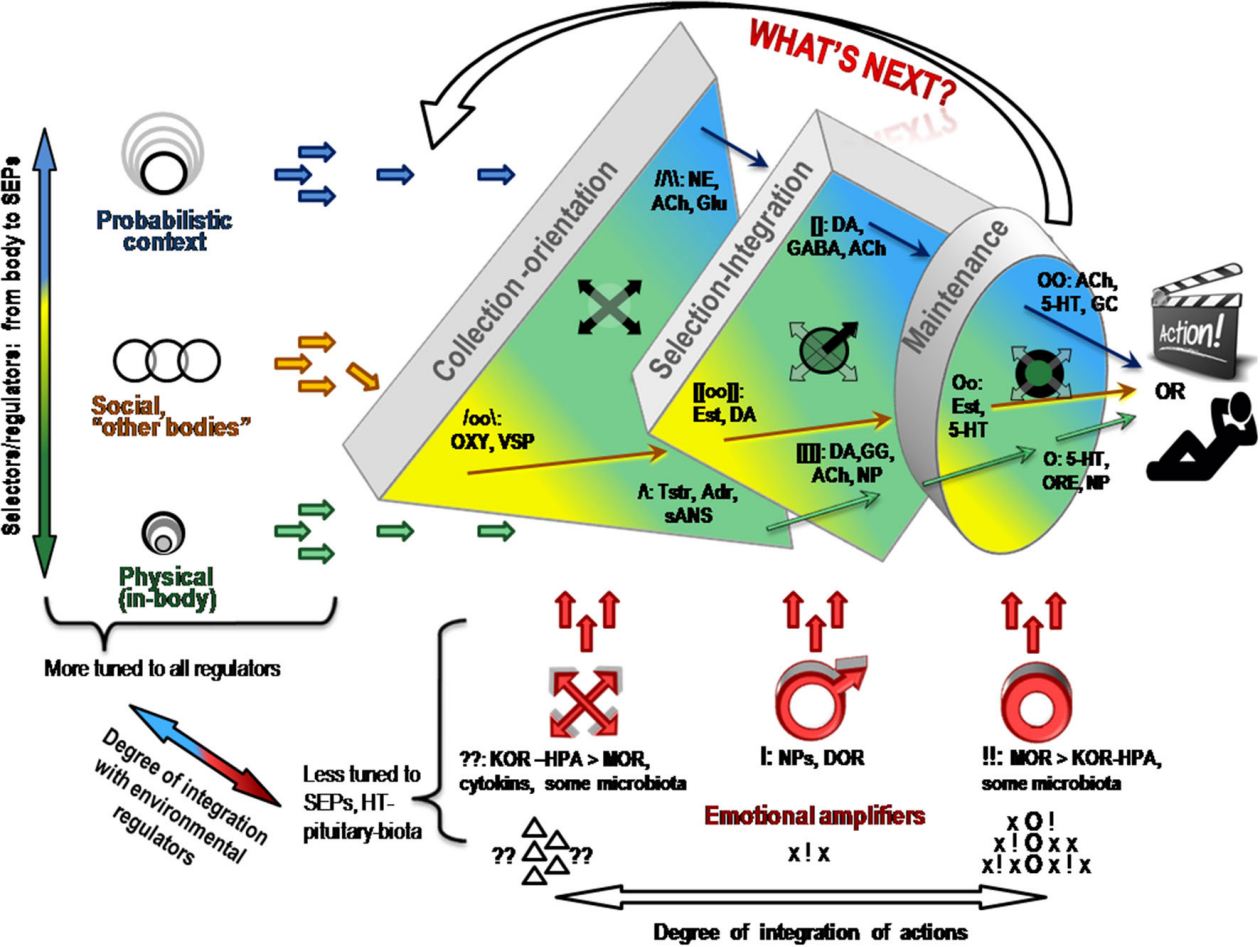
Neurochemical model Functional Ensemble of Temperament is presented here as a progression in the selection of behavioral alternatives in three approximate stages. There is an interaction between neurochemical teams within and across these stages. Emotional amplifiers coincide with the functionality of each stage. Ach, acetylcholine; NE, noradrenaline; 5-HT, serotonin; DA, dopamine; OXY, oxytocin, VSP, vasopressin, Tstr, testosterone; Adr, adrenalin; GC, glucocorticoids; ORE, orexins; NP, neuropeptides; Glu, glutamate; GG, GABA and Glu; OR, opioid receptor systems.

Let us return to the 1st evolutionary principle mentioned in the section Functional constructivism. Similarly to evolution, behavior is a result of multi-stage and multi-factorial natural selection of elements of actions. Contrary to the principles of behaviorism, stimuli *per se* are not the starting stage of behavior but are requested by the body, whenever body (including brain) is ready to deal with them. There are too many stimuli around, and there are too many ways to react to the same stimulus. Indeed, the experimental work of Bernstein (29–31) and Bartlett (32) in the mid-1930s demonstrated that behavior is a result of a selection from multiple alternatives (“degrees of freedom,” or d.f.) in perception, attention and elements of observable actions. Bernstein was credited for describing the *degrees of freedom problem* in motor behavior (29, 30). The problem refers to the fact that the same result can be achieved by multiple trajectories through moving parts of the body; vice versa, the same motions can be employed in reaching different results. A similar excess of d.f. was noted in neuroscience, in the behavior of neuronal ensembles and in the multi-chemical processes during the neurotransmitter release. When highlighting functional differentiation between neurochemical systems, the FET follows the functional stages of selection of d.f. in construction of behavioral act (Figure 3).

The SEP concept discussed above complements the view of behavior as a product of mutual selection between an individual and environment. This interaction includes the choice and arrangement by the individual of specific environment for more compatible functioning. Thus, the features of the environment and tasks that people prefer reflect their neurophysiological capacities. Hence, in our search for taxonomies of neurochemical biomarkers and bio-behavioral traits, it is useful to look for taxonomies and universal features of behavioral tasks and activities.

A first type of universal features of behavioral tasks relates to *orientation*, a first stage of selection in the ocean of degrees of freedom. Neurochemically, there is a team, consisting of noradrenaline (NE), GABA-Glutamate, acetylcholine, Neuropeptide Y, Substance P, stress hormones and kappa opioid receptors systems that is activated in novel or complex situations when an individual doesn't already possess relevant integrated behavioral programs. The same system is activated when the set of known or learned d.f. in behavior is not sufficient for the integration of future actions (i.e., when the nervous system needs to look for additional d.f.) We label this “Orientation-expansion” team as 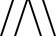, to resemble a triangle as “alert” symbol with a double (cortical) control.

The brain's NE systems have been linked to cognitive arousal, orientation and attention to novelty (68–70), supporting the idea of the “expansion” and “exploration” functionality of these systems as suggested by many authors (24, 28, 68, 71–73). The brain's NE system is most active in stress, in darkness, in tasks requiring attention to fast-changing situations and orientation, especially the occurrence of unexpected sensory events (68–75). The response of NE neurons to novelty is so specific that when previously novel threatening stimuli are presented repeatedly, they gradually evoke less and less NE neuronal firing (75, 76). Experiments on rodents showed a key role of NE networks in the PFC in attentional set-shifting tasks (69, 70, 77), and a NE deficit was linked to difficulties in learning new information (69, 71, 73, 77, 78).

There is a strong NE-ACh entanglement in attention processes, in which alertness to novelty is provided by NE modulation whereas sustained attention (i.e., monitoring the changes in more slow, steady context) is regulated by BF-cortical ACh (72, 74, 79, 80). In the cortex, both NE and ACh use volume transmission, and there are more subtle levels for their mutual regulation (4, 10, 11). ACh is a key player in the probabilistic processing system. It modulates the potential within multi-layer cortical Glutamate-GABA (GG) systems, whose vertical and horizontal connectivity generates anticipatory models of perceived features of the environment (15, 36, 62, 81–85). Since most work on information processing is done by neurodynamics within the GG networks, they could be also regarded as key players in probabilistic processing. The ACh-GG cortical networks provide the processing of “here and now” comparing it to “what is new,” i.e., novelty highlighted by NE-Glu networks (72, 74, 86). The fundamental role of GG systems, mediating brain ACh- and monoamine-based neurotransmission is seen in several types of mental illness (87).

Neuroanatomically, BF cholinergic neurons receive ascending brainstem input from adrenaline containing neurons of the medulla and NE neurons in LC, and synapses with afferent from AM and HT nuclei (88–91). The interaction between these systems continues at the cortical level, which is diffusely innervated by the BF's Meynert nucleus and where both ACh and NE commonly use volume transmission. The selective and tonic influence of cortical ACh activity assists in early stages of goal-setting and probabilistic estimates for possible successes, failures and other potential outcomes of events.

The “Orientation” system is always involved in monitoring the familiarity of environment and events and, therefore, is active, to some extent, even in familiar situations. In situations of novelty or uncertainty, brain NE systems can abruptly interrupt the activity of neural networks and re-organize them, facilitating rapid behavioral adaptation to changing contexts (92). Noradrenergic locus coeruleus (LC) and cholinergic ldTA and PPN nuclei in the brainstem project widely into both–autonomic and central–nervous systems (70, 78, 88, 93, 94). This allows the NE system to be a key player in Fight-Flight-Freeze behavior when the imbalance between needs and capacities related to the situation is extreme, and capacities to address this imbalance are not immediately available. In challenging situations, the 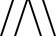-System uses its direct management of the sympathetic ANS and the HPA axis that prepares the body for drastic differences in behavioral alternatives: changing the heart rate, blood pressure, suppressing digestion and elevated muscle tone. The FET, therefore, combines the sANS and HPA axis to a regulatory System of a lower level of a more rough of expansion of behavioral alternatives (labeled here as ∧-System, to resemble a triangle as “alert” symbol without cortical control).

When the cortically-driven 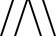-System of orientation underperforms (as a result of dysregulation of MAO inhibitors or other factors), the hypothalamic-pituitary, adrenaline-based ∧-System takes over inducing inability to focus and high impulsivity, known in ADHD. In combination with high testosterone (95, 96), low cortisol (97) and fluctuations in adrenaline levels, individuals with this adrenal under-arousal, high testosterone and low cortisol might develop CBPs know as Sensation Seeking (SS) (98–101), including dispositions for addictions (101, 102). The Sensation Seeking concept relates to behavioral orientation to stimuli or activities that could increase HPA arousal and does not necessarily includes novelty seeking (NS) or rewards seeking. The NS involves more NE networks and element of novelty processing, and the RS involves more DA activity and integration of actions. SS has a strong sex dimorphism (males have several times more SS-related accidents than females) (103) implying a role of testosterone in SS. In contrast, sex difference in DA are not as dramatic as for testosterone, and they couldn't explain sex dimorphism in SS and addictions unless explanations are coupled with the NE systems (102, 104). Besides, the SS is associated not only with the male sex but also with younger age (103), and the rates of decrease in SS coincide with the rate of decrease in testosterone but not in dopamine.

Another type of behavioral orientation associated with distinct hypothalamic-pituitary hormonal systems is empathy (labeled here as 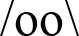, using the “oo” symbol for a between-individuals regulation). Empathy was most strongly linked to oxytocin and vasopressin systems (58, 105–108), and to their reciprocal interaction with gonadal hormones, especially testosterone (109, 110). Two distinct parts of the pituitary (anterior and posterior), show, therefore, at least two distinct types of behavioral orientation (SS and empathy) competing with a third type of behavioral orientation dominated by cortical control and probabilistic processing (58).

### Three Types of Behavioral Integration: Automatic vs. Novel/Complex vs. Impulsive

Behavioral orientation is a first, rough stage in the selection of d.f. in behavior driven by the body's needs and capacities and by the SEPs requirements. The second stage of selection, with much stronger trimming of degrees of freedom is performed by frontal—ventral-striatal systems and supervised by dopaminergic control. This stage involves the integration of both established and never-tried d.f., the choice of what to implement in action, prioritizings and sequencing them for future action. Early constructivists, such as Bernstein (29, 30) and Anokhin (33), and subsequently the whole disciplines of cybernetics and kinesiology, called the process of integration *programming*. The final choice of what direction, for example, a hand should move to, or what words should be said, out of the massive number of potential trajectories for hands and words, plus the sequencing of these actions in a specific order—the “program” of this action—depends on the position of the body, the position of the object, the intentions of the individual, multiple contextual variables and the properties of the object itself.

Programming, labeled here as [] (to resemble a check-list symbol), is very close to the concept of planning except, unlike planning, it is often unconscious or subconscious in nature. The ability to re-program behavior under changing situations is called *plasticity*. However, as is commonly known, behavior, to a large extent, is generated using previously tried elements—trial and error, habits, skills, attitudes and knowledge. When trial and error is employed, people learn how to suppress a massive number of irrelevant d.f. in actions and proceed with the final few most efficient d.f.

During learning, they develop a set of possible responses related to every skill, becoming ready for a variety of situations. Let's label these learned and established sets as functional [[]]-units, to resemble a double check-list symbol. The more that a person is familiar with the units of actions, the faster these actions can be integrated. This speed of integration of learned actions is known as *tempo* (e.g., motor-physical tempo or tempo of speech). Most often, behavior is being constructed on a continuum between [[]] and [] [i.e., [[]] 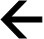 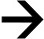 []], when it involves recombination of learned [[]] skills during repetitive, stereotypic actions, or just small trials of something new, with moderate demands for plasticity []. When an action is being constructed, the previously learned (habits) actions have priority for being chosen, due to their ease of integration and low need for orientation. For example, when faucet breaks in the kitchen, calling a family member for help is much easier than looking for a plumber because it is often the most learned d.f. at that moment (even though the “plumber” option might be more efficient).

Here is where the 3rd evolutionary principle listed in the Section Functional constructivism, appears to be useful. Earlier we named the mechanism of the development and use of pre-fabricated subsystems “cruise controls” evolution (23, 59). In this context, Bernstein's experiments in the 1930s, which pioneered the constructivism approach, deserve one more credit. In addition to the concepts of action's construction, degrees of freedom and the generative nature of the behavior, Bernstein demonstrated that there are likely several *levels of control* over the construction of action. With learning, this control is passed from the upper levels [i.e., more conscious []-integration] to lower levels [automatic, [[]]-integrations] but, when the construction of action faces challenges, it shifts back from automatic [[]] to more conscious [] levels. The preservation of learned units of behavior is known as habit formation that helps to optimize, ease and simplify behavioral programming. Habit formation takes the load off of conscious processing and transforms learned elements of cognition or action into ready-to-use habitual [[]] units, which, similarly to cruise controls in driving, require just initiation for their use but no longer require new orientation and programming.

Finally, in addition to behavioral integrations that adequately select elements of actions according to context and features of objects, there is one more form of “fast and dirty” integration: when there is strong HPA arousal, that has the power to suppress normal contextual processing. In this case, a behavioral integration (called *impulsivity*) is driven by fast-acting stress hormones, overriding the slow action of brain neurotransmitters. Coming back to our faucet example, if it breaks suddenly, with a loud noise or a massive flood of water, this might activate the HPA (Flight-Fight-Freeze) response, with a corresponding rush of stress hormones into the blood. A person might impulsively hit something, appropriately yell at their partner or react to absolutely unrelated stimuli with aggression.

It is important to underline that the “construction,” or generation of behavior is a mainly unconscious process, and the integration (or “programming”) of behavior an on-going gradual “calibration” of actions in tune with the properties of the situation and surrounding objects. In this programming, there is a narrowing of existing d.f. but never to a single Big Plan. Instead, multiple options end up in the final “program limiting these options to a small set (Plan A, Plan B, Plan C, and a No-way-Plan). This multiplicity of options for each action ensures adaptability of behavior to various contingencies. Only when subconscious recombination of pre-learned units of actions is insufficient to meet the needs of a situation, an orientation (i.e., a search for new d.f.) is activated, and actions become more conscious, and the plasticity uses more cortical resources.

### Neurochemical Biomarkers of [] 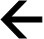 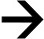 [[]]- Integration Systems

The most prominent neurochemical ensemble regulating the integration of actions (i.e., choice of d.f. and their sequencing) includes dopamine (DA), acetylcholine (ACh), Glutamate and GABA (GG) systems, with the support of several neuropeptides, all contributing something special to the process of behavioral integration. There is, as noted, a diversity of receptors within each of these systems, with differences in their functionality. Here, however, we focus only upon general patterns of functionality of NT these systems, setting aside their receptor-related differences.

Dopamine is often regarded as a “neurotransmitter of pleasure” and positive affectivity; however, there are numerous examples showing that this is not always true (111–114). In fact, negative stimuli and situations enhance activity in the mesocortical DA system to a higher degree than do positive ones (113, 114). An excess of extracellular DA was linked not to more positive moods but to schizophrenia (115–117), psychoticism (117–119) and, when combined with a 5-HT deficiency, to rigidity of behavior in OCD (120–123). The association of dopaminergic VTA-NAc projections with positive moods and motivational processes could be largely explained by the high involvement of opioid receptor systems in these brain structures.

Experimental studies have linked DA systems to a spectrum of functions that are unrelated to positive or negative emotionality but instead are related to the prioritization of behavioral elements (i.e., the key feature in behavioral integration). The DA system doesn't work alone in this process and uses ACh and GABA-Glutamate systems as mediators. In fact, the DA system is much smaller than its partners but works as a modulator of other systems, when it comes to plasticity, tempo, impulsivity and the assigning of significance to perceptual elements. There are differences in the work of these teams related to different integration types, suggesting that we should probably consider them as separate CBPs:

1. Novel integration or re-integration (change) of actions in changing, complex or unknown situations [plasticity of behavior, []], was linked to the interaction between DA mesocortical and mesostriatal networks (28, 124–127). In the caudate nucleus, DA and ACh release is complemented by 5-HT release from projections from the dRN to many cortical areas (123, 128). The tonic arousal from ACh release and the energetic supply from 5-HT is used to either trim or highlight signals emerging from multiple potentials of the Glu-GABA (GG)cortical pyramidal neurons. ACh-GG cortical networks, therefore, play a key role in behavioral plasticity, allowing for the simultaneous activation and editing of several scripts of actions (124–127, 129, 130), prolonging the availability of these scripts and having multiple options available for relevant combinations.
2. The stereotypic integration of suppression-excitation of pre-learned [[[]] - pre-fab] motor skills needed for routine activities employs DA in the striatum (126, 131–136), cholinergic PPN (137) and cerebellar (138, 139) networks. In the striatum, DA modulates the activity of ACh interneurons, selectively suppressing ACh release according to priorities in actions. In the striatum, the ACh interneurons and GABA inhibitory neurons basically are the striatum, as they represent over 95% of striatal cells in humans (132, 133), and DA is the lead modulator of these cells. The cholinergic and DA-ergic systems in the striatum are mainly inhibitory: integration of a program of actions requires extensive trimming (i.e., suppression of multiple d.f. in behavior in favor of the final few trajectories of actions). The support of serotonergic projections from RN to the cerebellum (139) and part of the striatum provides another sustainability component of the learned motor elements.
3. It has been consistently shown that with the increase in action program certainty, habit formation and more automatic integration (deterministic conditions), control over the integration of action is passed from the cortex to ventral striatum, then to dorsal striatum and to cerebellum. Vice versa, with an increase of task complexity (probabilistic condition), control over integration is passed from dorsal to ventral-striatal-cortical networks (131, 132, 134, 136, 140, 141). DA/ACh regulation in the dorsal striatum contributes to the shaping and sequencing of actions, which is complemented by parallel cerebellum-thalamic and RN-cerebellum networks for more automatic motor control, such as for posture and balance (139). This resembles Bernstein's theory of multi-level control over the construction of action (29–31).
4. The spontaneous, impulsive type of behavioral integration was also linked to DA release (142, 143). It has been shown that unlike in plasticity, habit-based behavioral and impulsive types of integration both involve lesser cortical activity. However, unlike tempo, impulsivity emerges when cortical monitoring is compromised (as seen in ADHD) (124, 125), with more involvement of hormonal systems (97, 144).
5. In cognition, DA networks are involved in assigning significance and priorities to stimuli (saliency). This is noted for both negative and positive stimuli (111– 114), and so DA cannot be seen as a NT of positive-only emotionality. The DA role in salience attribution is seeing not only in schizophrenia (115–117) but also in delusional components of the OCD (145).

The []-system of integration briefly sketches the (mostly unconscious) plan of performance for the current situation. Most [[]]-units comprising this plan will consist of learned previously units though from different contexts. The actual trajectories carried out by the resulting actions will depend on many environmental and somatic factors, and necessitating adjustments that occur simultaneously at multiple scales: from the office layout down to the movement of fingers. Through iterations the “main plan” becomes ever more consolidated, improving accuracy through transitioning control over the action from ACh-GG-DA cortical networks to the ventral striatum and then dorsal striatum networks (131, 134, 136, 140, 141).

Another putative neurochemical player in the ensemble that regulates the integration of a behavioral act is the delta-opioid receptor system (DOR). The highest DOR density was observed in the ventral striatum—NAc, caudate and ventral putamen—exactly those structures which were linked to the generation of behavioral programs (4, 146–148). DOR, as well as mu-opioid receptors (MOR) facilitate DA release in the striatum (149–151) speeding up the integration of automatic actions. The FET model suggests that the assistance of DOR system is speeding up actions, under the condition of strong HPA arousal and diminished cortical control, thus serving as an emotional amplifier of impulsive integration of behavior.

There seems to be a division of labor between the two catecholamines, NE and DA. If the NE –Glu team is the lead modulators of orientation to novelty in behavior, then the DA-GABA team, with the support of ACh, are the lead neurotransmitter team in the integration of actions, and there are almost antagonistic relationships between these two systems. Neurochemically, NE and DA represent the same catecholaminergic system sharing one pool of regulatory peptides (152). In the cortex, NE controls the release of the DA as most cortical DA comes not from the DA-producing neurons in the VTA and SN but from NE neurons, and, after release, DA is recaptured back by NE, and not the DA transporter (152, 153). In the striatum, however (i.e., the areas that are involved in the preparation of motor programs, and selection of action), NE does not get involved in further []-[[]] selection of behavioral elements. In fact, the NE projections from the LC mainly avoids the striatum but are abundant in the areas associated with attention and sensory processing (thalamus, parietal cortex, the pulvinar nucleus, the superior colliculus and AM) (92–94). Therefore, as the integration of actions becomes more and more determined and less probabilistic, progressing from cortical [] “sorting” to dorsal striatum [[]] DA-led systems, the involvement of NE becomes less significant.

These neurochemical and neuroanatomic differentiations between the NE and DA systems indicate their differences in functionality in the construction of behavior. These two systems literally handle the choice between (1) stopping the behavioral integration and orienting for additional alternatives, or (2) stopping the expansion of alternatives and continuing with the integration using known options.

## Body Bias and Context-Dependent Maintenance of Behavioral Alternatives

### O-System of Pro-body Homeostatic Maintenance and the Embodiment Principle

If a situation doesn't require re-integration of actions, behavior can be driven just by previously integrated routines or by homeostatic maintenance (such as in rest and relaxation) with very minimal attendance to external stimuli (first stage of action in Figure 3). Since it is the body that performs the behavior, a selection of d.f. is normally tuned to the body's needs and capacities. Without either need or capacities (including those of the nervous system), the optimal behavior will not be integrated. Termination of behavior also occur when either needs are met, or capacities are expired. In this sense, the body is probably the final and most powerful selector of behavioral alternatives, out of all of the selector-systems discussed in this paper.

All neurochemical processes underlying the homeostatic maintenance of behavior are operating in the form of interconnected cycles of composition and decomposition of chemical components, so we use the notation “O”-system for the neurochemical team described in this section. The leader of this team is 5-HT systems (154–156). Serotonin is not stable and must be manufactured by the 5-HT neurons from tryptophan before its use and then quickly decomposed, to be manufactured again, under the condition that there is a supply of tryptophan. Ninety percent of tryptophan 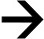 serotonin is used by the body, not the brain for the regulation of many internal organs, including blood vessels. In this sense, 5-HT is very much a “pro-body neurotransmitter.” In comparison to other neurotransmitters, the brain makes only a very modest amount of serotonin, and 5-HT-neurons in the raphe nuclei (RN) represent only one-millionth of the total population of neurons (154, 157). However, these small groups of neurons could be considered as a maintenance-CEO of the brain: they project to almost every brain structure and receive inputs back from dopaminergic SN, VTA, noradrenergic LC, cholinergic superior vestibular nucleus and epinephrine nucleus of the solitary tract. The RN also projects and receives inputs from the hubs where all four neuromodulators meet: the hypothalamus (HT), amygdala (AM), hippocampus (HC), and cortex (154–158).

Serotonin release was linked to multiple regulatory features, which have common functionality, namely the control of the optimal boundaries of the metabolic cycles associated with sustained performance. For example, in the body, 5-HT is in control of both expansion and contraction of blood vessels (155, 156). In monitoring food consumption, 5-HT innervations to the hypothalamic paraventricular nucleus (PVN) tunes it to the needs of the organism, preventing over-eating by modulation of gastric activity, controlling macronutrients in the diet and influencing responses to the gustatory quality of food (154, 155, 157) and most likely regulating the action of social hormones in the brain (159). Sudden demands for changes in behavior and novel actions decrease 5-HT firing, and vice versa—there is an increase of 5-HT neuron firing in stereotypic actions as chewing, sucking, walking, painting, maintaining physical posture, etc. Firing of 5-HT neuron inhibits alertness and information processing by afferent systems (75, 123), consistent with the idea that the maintenance of routine actions and orientation are managed by different networks.

Serotonergic transmission often doesn't use permanent synapses and uses volume transmission, especially in cortical, forebrain and basal ganglia areas. However, when it comes to 5HT regulation of the hypothalamic wakefulness control, the 5-HT system is less fuzzy and much more organized (154, 157). Innervations from 5-HT-gic RN hold several HT nuclei “by the neck,” having well-defined and dense synapses, for example, to the suprachiasmatic nucleus (SCN), a manager of circadian rhythms. Melatonin (a sleep-inducing hormone) is synthesized directly from 5-HT, and the pineal gland contains all of the enzymes necessary to synthesize 5-HT from tryptophan as well as two additional enzymes required to convert 5-HT to melatonin. This allows the 5-HT system to directly moderate and inhibit the impact of excesses of light on circadian activity but maintain the daily rhythm of corticosterone for optimal arousal and sleep when activity is not necessary. There are also direct synaptic connections between 5-HT terminals and CRH-containing neurons in the PVN of the HT.

Another embodiment system cosists of neuropeptides (NP) (including fast neuropeptides—hormones), which are regulated by the hypothalamus (HT), the center of cooperation between the endocrine and nervous systems. Namely neuropeptides and not other neurotransmitters are the most common NTs in the HT. The HT manages energy metabolism, endurance, adenosine-based arousal and orexin-based wakefulness, appetitive and other homeostatic functions (6, 24, 28, 160–162), affecting individual differences in energetic capacities. The HT is also the key regulator of the autonomic nervous system (ANS) that innervates all body organs and employs two other neurotransmitter systems, NE for the sympathetic sub-system and ACh for the parasympathetic sub-system. NP systems are essential co-releasing factors in neurotransmission for all monoamines (4, 160–162). Their interaction with other NTs emerged as a factor in individual differences in CBPs (97, 144, 162).

The third embodiment system, gut microbiota, generates practically all neurotransmitters found in the human brain, including monoamines, acetylcholine and peptides that bind to opioid receptors (163). Moreover, neither brain nor body make their own tryptophan with both relying on tryptophan supplied by gut microbiota or, to a lesser extent, by food. This makes gut microbiota an important player in the embodiment network (163). Almost of all these biota-made neurotransmitters are used locally, to regulate the “behavior” of internal organs (gut motility, smooth muscle contractility, glandular secretion, glial signaling etc.). Previously, biological psychologists paid little attention to these microbiota's NTs because the blood-brain barrier prevents these NTs from directly influencing the neurons. Now we know that gut microbiota do influence brain function but indirectly, via the HPA axis and glial cells (163–165). A small portion of microbiota derived tryptophan is shared with the brain for the final transformation to serotonin (i.e., a key neurotransmitter of the O-System), but this process is still limited by having only one chemical transporter which multiple neurotransmitter systems must share to reach the brain cells (6, 155).

A fourth embodiment system that is involved in homeostatic maintenance of behavior and associated psychosomatic processes is the immune system. It contributes to the development and plasticity of synapses through regulation of many NT systems. Immune cells express NE receptors, respond (non-synaptically) to ACh, produce endorphins, affect opioid receptors binding, contribute to the production of serotonin and brain/pituitary peptides, and have mutual regulation with the HPA axis (166–169).

Finally, glial cells are recognized as “service stations” to neurons and as a middle-man between brain cells and blood vessels. The fundamental role of glia cells in neuronal life begins with the birth of neurons, when they migrate along glial cells and axons to reach their final position. Radial glial cells serve as precursors to neurons in the brain and provide a scaffold for their radial migration [(170), p. 1190]. Microglia cells provide repairs of synaptic connections, respond to injuries and infections and also monitor the electrical activity of neurons (171, 172). Glial astrocytes provide homeostasis and regulate neuronal excitability and overall neuronal plasticity (171, 172). Moreover, the most prevalent neurotransmitter in the brain (90%+), Glu is synthesized not by neurons but by glial cells (astrocytes) and then transported to and from neurons by a special transporter. Finally, glial astrocytes, in contrast to neurons, have access to blood capillaries and so are not cut off from blood borne influences by the brain-blood barrier as much as neurons. This means that the state of the blood (reflecting body's physiological processes) can affect glia, and this is in fact what happens during infections or exposure of the blood to toxins, such as alcohol or infections. The increase of cytokines during infections (167) or toxic metabolites from processing alcohol (173, 174) cause inflammation of glial cells, compromising their functioning, and this directly compromises neurons' functioning.

The contribution of 5-HT, hypothalamic NPs regulating endocrinal functions, involvement of microbiota, immune and glia systems in the O-team of behavioral regulation provides for maintenance of the optimal range of metabolic activity of both body's and brain during behavioral construction, preventing their over-use or under-performance. This functionality can generate a “pro-body bias,” or an embodiment in estimations of the needs and capacities of the individual. This can explain the results of our experiments investigating the impact of the endurance-related temperament traits on semantic perception (159, 175–177). Participants with lower physical and social endurance in our experiments estimated abstract neutral concepts in less positive terms than participants with higher physical and social endurance. In other words, individual differences in endurance systematically affected meaning attribution (i.e., highest cognitive processing of individuals) and likely their decision making. In 1995–1999, when we discovered this phenomenon we called it “projection through capacities” but nowadays we use a more compact term for it—embodiment. The effects of body biases on cognition, behavioral choices and decision making, known as embodiment, are now well-known in the literature (178).

### ACh-5HT Based OO System of Contextual Monitoring

If the O-System produces the pro-body bias in the selection of behavioral alternatives, the neurochemical team described in this section, takes care of the selection of d.f. related to the current environmental context (i.e., providing a pro-SEPs bias). The FET highlights the leading role of ACh system in this team, in line with the strong consensus in neuroscience about its role in sustained attention, cue detection, context processing, and memory (89, 179, 180). Sustained attention actively monitors established situations whereas attention to novelty is activated when established “labels” to the situational elements don't fit (86, 89, 180). Projections from the cholinergic cells in the basal forebrain and cortical interneurons dominate the cortex, hippocampus and cerebellum (i.e., brain areas that are most associated with cognitive functions) (90, 179, 181). They have extensive axons building up horizontal cortical inter-connectivity (62, 89, 158, 179) combined with bottom-up (“vertical”) projections from the thalamus to the cortical layer IV (182). This interconnectivity facilitates a simultaneous “cross-talk” between different cortical areas and layers during the cholinergic modulation of excitation of pyramidal neurons. ACh partners with GG systems in the provision of sustained attention (81, 82, 183).

The ACh system also combines the well-defined topographic segregation of BF projections (91) with overlaps in these projections on specific target cells (62, 158, 182) highlighting cross-cortical modulation of sensory gains. These ACh projections can adjust the size of perceptive fields, and so change the focus on specific details of the situation (184). With repetitive training, the responsiveness of neuronal groups that processed particular properties of the context enhances, and the long-term modulation provided by mACh receptors helps to strengthen the lateral connectivity between similarly tuned neurons (182). FET labels the system as OO, to highlight the fact that maintenance of sustained attention proceeds as interaction with environmental cyclic processes.

The 5-HT systems in frontal, entorhinal and cingulate cortices assist ACh activity in contextual information processing. These regions have the highest 5-HT presence out of all cortical areas (4, 128, 154, 155, 157). A high density of 5-HT terminals and volume transmission of 5-HT in the frontal cortex complements the tonic arousal of the OO-System (that monitors small adjustments of behavior). There is a high density of ACh projections in the OFC (62, 83, 89, 182), with extensive volume (extracellular) transmission of both 5-HT and ACh (8, 10, 11) suggestive of mutual regulation of cortical O-OO Systems. This mutual regulation is not limited to the cortex as seen from the cholinergic PPN-hypothalamic projections, projections from ACh neurons in dorso-lateral tegmental nucleus (dLTA) to the RN and from the RN to HC, BF and the cortex.

Several sources note that ACh-producing cells contain two distinguishable forms of storage of ACh (6). Only one of these forms is readily available for release (“depot pool”) as it consists of vesicles positioned near the plasma membrane of the axon terminal, rapidly responding to axonal depolarization. The second (“reserve” or “stationary”) pool, is likely present in more distant vesicles and refills the depot pool as it is being used. However, the reserve pool is used truly as the last resort as the vesicles are refilled first with the newly synthesized ACh from the depot pool and only then use the reserve pool for the refill. This double-storage feature and long-term potentiation/depression (LTP/D) gating (62, 83–85) allows ACh systems to play a key role in sustained attention and probabilistic processing of events before, during and after their unfolding. Another feature of ACh storage contributing to its role in sustained attention is the use of a neurotrophic factor that supports cell functioning and that changes very slowly in ACh cells (158). The ability of ACh to persist longer within a cell and vesicles without being decomposed and the double-storage mechanism likely contribute to its key role in memory, learning and attention.

Gathering information about probabilities of events and their processing involves the memory function provided by ACh projections from the midbrain to the HC, HT and to the cerebellum (84, 85, 90, 137, 138). Since the HT gives a constant update about the present state of the body's needs and capacities, such a combination could create a holographic image of past-vs-present states of the body. Another set of cholinergic projections arises from the higher midbrain area to the thalamus, AM, latero-dorsal forebrain and cortex (i.e., brain structures producing a holographic impression of present-vs-future capacities, needs and environmental context) (137, 138). Such holography is convergent with the horizontal, cortical-to-cortical projections, allowing a past-present-future comparison in the assessment of the context of the situation and in developing a course of actions. The ability of ACh systems to model a timeline of events facilitates the analysis of their frequency, duration, changes, associated causes and outcomes. As has been shown in the experiments of Hasselmo's group, the same neurons in entorhinal cortex and HC encode both time and space in the construction of actions (180, 185). There are “split” neurons that are active in ambivalent contexts when a choice should be made and “grid” neurons that keep the information processing about the background context (180, 185). Overall, the brain connectivity of ACh neurons, functional segregation and task-driven overlap in ACh projections, volume transmission and intracellular double-storage mechanism give this system the ability for timely information processing, putting it into the context of the past events and future planning.

## Opioid Receptors' Up/Downregulation Can Generate Body-Based Emotional Dispositions (!I?-Systems as Emotional Amplifiers)

Endogenous opioid receptor (OR) systems comprise a class of G-protein coupled receptors (GPCR) regulating transmission between many brain NTs (41, 149, 186). ORs were considered first in the context of their direct effects on mood (pleasurable or analgesic) when they are administered to the body from external sources. Later it was found that the body is capable of producing endogenous binding agents (opiate peptides), and that the density of endogenous ligands is a rather plastic system. The density of OR ligands can be increased (upregulation) or decreased (downregulation and desensitization) depending on the supply of the peptides binding to them and the sensitivity of the receptors themselves. A single administration of opiates often triggers a set of changes that usually is restored by a chain of recovery mechanisms. Downregulation of receptors is observed mostly after chronic overuse/overproduction of these receptors' agonists as a protective feedback mechanism, while upregulation develops in cases of a persistent deficits of needed peptides (41, 149, 186, 187).

A detailed justification of the OR-related components of the FET model is given elsewhere (24–26). Here note that the OR are classified into four groups, three of which are most relevant here: mu (MOR) binding endorphins; kappa (KOR) reacting to dynorphins, and delta (DOR) binding enkephalins (41, 149, 186). These three OR systems (labeled here as “!I?”) likely affect the selection of actions inducing pro-body biases that are known as dispositional emotions, each contributing their specific aspects. The MOR system (labeled as !!) adds emotional valence to behavioral regulation: positive affectivity and a sense of approval of choices when the amount of endorphins is sufficient to bind existing receptors, and dysphoria when this amount is insufficient (41, 149, 188–192).

There is a strong coupling between MOR and 5-HT systems. When pregnant rats were chronically exposed to opium that causes downregulation of MOR receptors, they also had significant decrease in endurance in four types of maternal behavior, a decrease of 5-HT in HC, a decrease of BDNF and an increase of corticosterone (stress hormones) (193). Out of all OR only MOR develop heteromeric complexes with 5-HT1 receptors (194). Brain and guts' endorphins that bind to MORs have the capacity to suppress stress-related HPA arousal, KOR activation and NE release, thus providing a subjective feeling of security and the ability of an individual to cope with the current state of events (58, 149, 188–192, 195–198).

HPA-KORs (labeled here as ??) likely enhance perception of sensational and adverse features of the environment, experienced as elevated preparedness for perception (when the amount of dynorphins is sufficient to bind existing receptors) or lack of interest in it (when this amount is insufficient) (41, 186, 197–199). KOR system was indeed linked to chronic anxiety and perceptual sensitivity (103, 186, 197–200).

Finally, the action of DORs (marked here as “I”), originally linked to positive emotionality, appears to play a role in behavioral mobility, speed of generation of actions, including its premature generation (i.e., impulsivity) or compromised motor control (as in Parkinson disease). DORs and their binding peptides are highly present in DA-rich striatum and other basal ganglia (4, 147, 148, 150) (i.e., brain regions implicated in the generation of scripts and shapes of locomotor actions). DOR-MOR activation as an approval of the program of actions signals higher capacities and suppresses NE and cortical ACh but activates DA release. This might explain why planning where the options are certain is often accompanied by positive emotions and by activation of the limbic structures assigned to the “reward networks,” with a high presence of MOR (VTA-NAc). MOR-DOR heteromers in the striatum and other basal ganglia facilitate DA release (201) and, with the support of ACh interneurons, shape the program of actions. The DOR system likely contributes to speeding up of the integration of behavior, seen in impulsivity and tempo of actions.

Since binding peptides are produced internally, by microbiota and several sites in the brain that vary among people, the degree of imbalance between the supply of these peptides and their corresponding receptors' density can determine whether or not the CBP will emerge as a temperament trait or as a symptom of psychopathology (**Tables 2**, **3**, as example). Regardless of the degree, the dynamics of OR-activation cycles can be a factor in emotional behavioral dispositions that persist even in the absence of triggering events that could explain them. In light of these arguments, the FET model includes three OR-driven CBPs: Neuroticism (as KOR suppression of MOR systems and DA release), dispositional Satisfaction (as the opposite pattern) and Spontaneity (Impulsivity, as DOR dysregulation) (24–26).

## Putting it Together: The Functionality of Neurochemical Teams Seems to Match FC Partitioning

The brief descriptions of basic findings regarding the leading neurochemical teams illustrate that they all have different functionality related to specific spectra of behavioral d.f. that should be examined during the selection process. More importantly, their specificity appears to be logical, corresponding to the universal architecture of action construction described in many constructivism models (Tables 1, 2; Figure 3). This architecture includes orientation, information processing, programming and maintenance of chosen range of actions, with a feedback to the programming block for future adjustments. Individual differences in the construction of behavior can be, therefore, classified using at least three levels of contextual complexity and degrees of association with SEPs. These levels correspond to distinctions between physical, socio-relational and abstract-probabilistic regulators. The FET model organizes temperament traits and symptoms of mental illness in a 3 × 4 matrix categorized by formal functional aspects of human behavior (FET rows are highlighted in gray) [Table 2, Figure 3 (3, 24–26, 28)]. Nine out of 12 components within the FET represent traits regulating behavioral endurance, integration and expansion of d.f. (3 columns) at several levels of contextual complexity of behavior [i.e., physical, social and mental aspects of actions (3 rows of Table 1)].

**Table 1:** The neurochemical regulatory systems, with their notations and contribution to the development of the program (choice) of actions.

**Sign**	**System**	**Degree & type of SEP tuning**	**Degree of integration**	**Lead NTs**	**Support NTs**	**Universal features of situations, which are addressed by these regulatory systems & references**
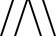	Orientation, probabilistic processing	High Wide contexts of SEPs	Low	Glu, NE	ACh, GABA	Novelty, complexity, uncertainty of situations; implicit information requiring probabilistic processing of events, whether occurring or possible; 1st stage of selection of d.f. for actions (24, 28, 68–73)
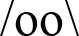	Orientation to others vs Self	Moderate Other individuals	Low	OXY	VSP; low Tstr?	A state (and a presence) of other individuals, including peers, offspring, parents, society members, imagined characters and animals (105–108)
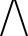	Fast expansion of body's d.f.	Moderate Body's response to sudden SEP challenges	Low	Tstr, Adr sANS	low cortical NE? low cortisol?	Urgency in orientation to situations requiring a fast suppression of programs that are too extensive for the current timeframe; a search for simpler behavioral alternatives and expansion of body's capacities. (95–102, 104)
[]	Program integration	High Wide contexts of SEPs	Moderate	DA, ACh	5-HT, GG	Multiplicity of d.f. in situations. This []-System helps framing the choice and sequencing of d.f. based on body's needs/capacities and information about SEPs received from 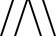 , 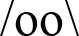 and 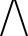 . This programming process considers several Plans (A, B, C, D…) (28, 124–127, 129, 130)
[oo]	Exe-verbal units of actions	Moderate Other individuals	High	DA, Estr	GG	Multiplicity and simultaneity of d.f. in verbal activities. This System regulates further selection of verbal actions, finalizing the Plan A, with a shadow Plan B, based on available executive resources of the social Oo system and SEPs demands (97, 103, 144)
[[]]	Exe-physical units of actions	Moderate Physical objects in SEPS	High	DA, NP, A	ACh, GG	Simultaneity of situational challenges requiring physical actions to be automatic and well-learned, to free attentional resources. This [[]]-System regulates further selection of physical actions, finalizing Plan A, with a shadow Plan B, based on avaliable executive resources of the physical O-System and SEPs' demands. Previously composed units of behavior have a priority for initiation during similar actions (126, 131–137, 140, 141)
OO	Context monitoring	Moderate Current context	High	ACh, 5-HT	GG, GC	On-going uncertainty with several but predictable (not novel) outcomes. This sustained attention System monitors specific targets, so actions are limited to d.f. of “here and now” (62, 81, 82, 90, 158, 179, 181–183)
Oo	Sociability	Moderate Other individuals	High	5-HT, Estr	OXY, H	Situational demands for prolonged and/or intense verbalization. This System, on average more advanced in young females, than in other groups, provides maintenance in socialization and other verbal activities (97, 103, 144)
O	Homeostatic maintenance	Low Physical state	High	5-HT, ORE	NP, H, gut biota	Situational demands for prolonged and/or intense physical endurance. This O-System, on average more advanced in males, provides maintenance of chosen actions in physical activities (154–158, 163–165)
??	Alertness—disposition	Low Novelty, uncertainty in SEPS	Moderate	KOR, cytokines, some biota	NE, GC, ACTH	“Red flags” in situations. This System enhances sensory mobilization sensitive to possible adverse outcomes when developing a program of actions. Prolonged mobilization creates an emotional disposition for negative expectations (neuroticism) (103, 186, 197–200)
I	Spontaneity, impulsivity	Low Initiation of actions in response to SEPs	High	Tstr, DOR > GABA	MOR, DA	Urgency in actions. This System facilitates spontaneity in integration of actions, approving initiation of actions without further 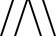 - and OO-based sorting. For that, DOR suppresses ACh release and works in line with the MOR-based !!-System (97, 124, 125, 142–144)
!!	Satisfaction disposition	Low Ease of approval of SEPs	Moderate	MOR, biota	5-HT, DA	“Green flags” in situations. This System enhances approval of selected d.f. in developing a program of actions. When prolonged, it creates an emotional disposition for positive expectations (satisfaction, optimism) (41, 149, 188–192)

*NTs, neurotransmitters; 5-HT, serotonin; ACh, acetylcholine; NE, noradrenaline; DA, dopamine; NP –some hypothalamic neuropeptides and hormones; Glu, glutamate; GG, glutamate and GABA; H, histamine; A, adenosine; ORE, orexins; (K, D, M)OR: kappa-, mu- and delta-opioid receptors correspondingly; HPA, hypothalamic-pituitary-adrenal axis; GC, glucocorticoids; Estr, estrogen; Tstr, testosterone; Adr, adrenaline; Cort, cortisol*.

**Table 2:** The neurochemical model FET (Functional Ensemble of Temperament, T-CBP), highlighted in italic-bold and the comparison of its component to clinical consistent behavioral patterns (C-CBP).

	** 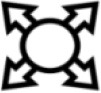 **	** 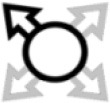 **	** 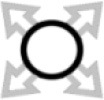 **	
**Behavioral aspect:**	**Beh. orientation & expansion:** 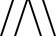 , 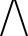 **,??**	**Speed of integration of actions: [], [[]], I**	**Maintenance (Keep) systems: O, OO, !!**	
**CBP type**	**Wide context, probabilistic, implicit aspects: MA, ACh, GG as leads**			
***T-CBP** **≈** **N***	***Probabilistic processing, PRO***	***Ease of change in actions: Plasticity, PL***	***Mental (intellectual) endurance, ERI***	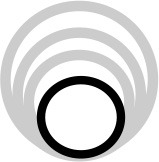
C-CBP < N	Low intelligence and comprehension	Rigidity (rituals in OCD)	Inability to focus as part of the ADHD	
C-CBP > N	Narcissistic PD? Part of schizophrenia?	Excessive start-ups without finishing them (e.g., in ADHD, mania)	Obsessions, as part of OCD	
**Social-Verbal aspects, tuning actions to other people: OXY, Estr as leads**				
**T-CBP** **≈** **N**	**Empathy, EMP**	**Social tempo, TMS**	**Social endurance, ERS**	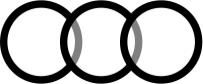
C-CBP < N	Autistic disorders	Expressive language problems	Social withdrawal	
C-CBP > N	Dependent PD	Mania	Histrionic PD	
**Physical aspects, determined by physical capacities: 5-HT, ORE, H and NPs as leads**				
**T-CBP** **≈** **N**	**Sensation seeking, SS**	**Physical (motor) tempo, TMM**	**Physical (motor) endurance, ERM**	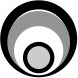
C-CBP < N	Generalized anxiety	Motor retardation and slowdown, Parkinson D.	Fatigue, sleep problems	
C-CBP > N	Antisociality, to bust low HPA arousal	Physical agitation	Athletic ability for endurance	
**Emotional amplifiers: OR, HPA, and GC as leads**				
**T-CBP** **≈** **N**	**Neuroticism, NEU**	**Spontaneity, impulsivity, IMP**	**Dispositional Satisfaction, SF**	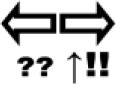
C-CBP < N	Indifference, detachment	Inability to be playful or spontaneous	Dysphoria, pessimism, low confidence	
C-CBP > N	Low tolerance to novelty/uncertainty, perceptual alertness	Premature integration of actions, behavioral reactivity, impulsivity	Too relaxed dispositions, over-optimism	

*Clinical patterns relate to cases of extremely high and low expressions of the given component, in comparison to the Norm (N). Bold font indicates the names of temperament trait in the FET. The labels of neurochemical systems described in the text: O, Maintenance; OO, Sustained attention, Contextual tuning; 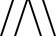, Probabilistic processing and orientation; I-∧, hormonal systems, “?, !”, Emotional dispositions; [], New Integration; [[]], Established integration. MA, monoamines; ACh, acetylcholine; NP, hypothalamic neuropeptides and hormones; GG, glutamate and GABA; H, histamine, ORE, orexins, OR: opioid receptors systems; HPA: hypothalamic-pituitary-adrenal axis; GC, glucocorticoids*.

The division into these three rows is known as the activity-specific approach (63–67). The functional specificity of cortical areas for verbal processing, abstract thinking and management of physical aspects of behavior, as well as the role of oxytocin and vasopressin hormones in social-affiliative aspects of behavior (105–108, 159) support this activity-specific approach. Moreover, there is a distinct segregation in hypothalamic-pituitary systems between cells manufacturing and responding to “social” hormones (such as oxytocyn, in anterior pituitary) and “physical” hormones (such as growth hormone in posterior pituitary, playing important role in physical capacities of the body). Interestingly that changes in estrogen are also associated with verbal capacities, as seen in superiority of young females in these capacities, in comparison to other age and sex groups (103). After a sudden drop in estrogen, as a result of menopause, it is mainly verbal, and not other types of memory are reportedly affected. This suggests the link between estrogen and higher ability for young females for prolonged socialization (named in the FET as Social Endurance), in comparison to males. The specificity of these systems can be traced to properties of the tasks of early humans when they faced high variable, diverse and complex environments, as well in development of societal structures. Activity-specific approach to the separation of CBP types appeared to be beneficial in both, temperament research and psychiatry (58, 64, 66, 67, 207–214).

The three emotionality-related traits [Neuroticism, Impulsivity and (a disposition for) Satisfaction] are emotional dispositions, linked to dysregulation of opioid receptor density. They amplify the three key regulatory aspects described in the FET columns: sensory-orientational mobilization; selective acceleration of actions and subjective comfort and security (24–26). A consistent dysregulation within these teamed relationships can induce behavioral dispositions to act a certain way (for example, either to have more orientation than performance or to have more performance than orientation; either have more verbal than physical behavior or vice versa).

The generation of behavior appears to follow the dievolution principle, described earlier, that highlights the multi-level iterative nature of emergent phenomena (23, 59). As can be seen from the functionality of neurochemical systems, the construction of an action starts from “many ends”—physical, social and mental capacities of individual and same types of expectations of environment—and proceeds as a compatibility-matching interactive process. After a rough sketch of possibilities, it goes through a process of natural selection of the most compatible options under the influence of multiple selection factors, progressing to the emergence of the final behavioral construct. If this construct appears to be useful, it is repeated within subsequent activities, “surviving” in the individual's memory and skill set, to be preferentially used later.

The 12 aspects of behavioral regulation discussed above are not “types” but aspects of actions, i.e., they are present in any action to some degree as a “holographic” space for the action. Moreover, each of these aspects is supported by related SEPs (i.e., environmental infrastructure), reinforcing this aspect (Figures 1, 2). For example, probabilistic processing is supported by science; sensation seeking—by industries providing alcohol, illegal drugs and risky amusements; empathy—by church services; plasticity—by jobs related to management and fast decision making; physical tempo and endurance—by athletic games; social temp and social endurance—by social media and infrastructure for fast and prolonged socialization; sustained attention—by demands of school systems and jobs such as accounting services or games requiring mental endurance (like chess or hunting); neuroticism—by jobs of controllers and inspectors; spontaneity (impulsivity)—by jobs like journalism; dispositional satisfaction—by jobs requiring strong stress resilience.

When individuals express their capacities, getting involved in such specialized SEPs, they promote these SEPs' industries. Thus, specific distributions of neurochemically-based capacities in certain communities affect specific distributions of industries in these communities. This might be seen as a functional differentiation “on a diagonal”: the economic and cultural infrastructures of communities driven by the neurophysiological (dis)abilities of its citizens, which, in turn, are based on specifics of the neurochemical systems of these individuals (and perhaps vice versa).

## Between Temperament and Psychiatric Disorders: Toward a Common Taxonomy

### What Can Possibly Go Wrong in This Complexity…

After highlighting the main “tea11ms” of neurochemical systems of healthy behavioral regulation, we can present the types of psychopathology in a more systematic and transparent way than current empirically-derived classifications of mental disorders. The neurochemical literature is extensive, and we barely scratched the surface of neurochemical complexity or interactions with environmental factors. One point is clear: there is more to neurochemical processes than signaling, excitation or inhibition. Almost all neurotransmitters have to be synthesized “on the go,” and, with the exception of ACh, cannot be stored for a long time. They operate in contingent cycles of constant production and metabolism. Therefore, when we refer to “deficiencies” in these systems, we mean dysbalances in these cycles (occasional over-production and under-production, or too slow/fast metabolic processes) rather than “deficient amount of neurotransmitter.” These processes are delicate, complex and constructive; many things can go wrong, and it takes a lot of adjustments to make things go right.

Application of an evolutionary perspective is essential when looking for classifications of functional aspects of behavior and to map the neurochemistry of its regulation. Our neurochemical “machinery” developed in evolution in line with the properties of the tasks that our biological species faced, such as demands for endurance, sustained attention in variable environments, probabilistic predictions for unpredictable situations, ability to face novelty and generate novel actions, societal processes and the ability to learn. In this sense, the functionality and partitioning of neurochemical systems that the FET model highlights are not arbitrarily chosen but correspond to specific functional aspects of human (and animal) activities. Similar to the universal anatomy of a healthy human body, that is used in medicine to sort out the nature of somatic illnesses, we advocate paying attention to the functional composition of behavior, when sorting out bio-behavioral differences. This functional composition is presented here symbolically, verbally and graphically, in Figure 3 and Tables 1, 2. Tables 2, 3 briefly illustrate how the FET framework and proposed formalisms can be used in classifying temperament traits and psychiatric taxonomies. Table 3 also cites studies that used FET-structure test (Structure of Temperament Questionnaire, STQ-77) on clinical samples. The STQ was introduced over 30 years ago (18, 63–65, 203), and its compact version, STQ-77 was adapted to 22 languages (17, 65–67, 202–204, 206, 215, 216).

**Table 3:**
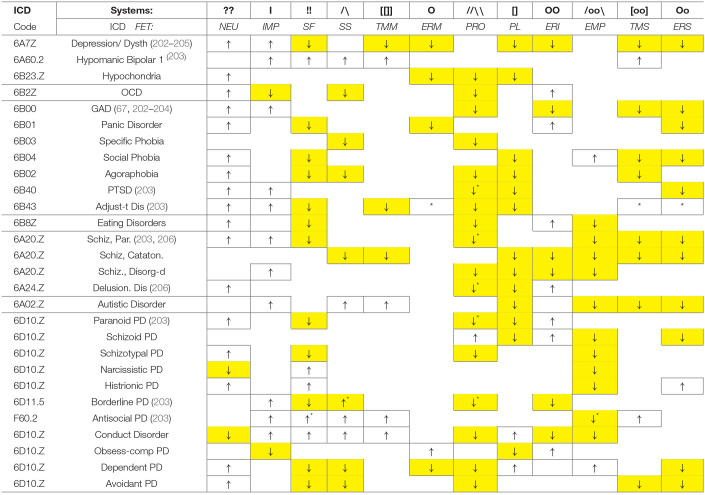
Proposed correspondence between described neurochemical systems, temperament traits of the FET model and main diagnoses of psychopathology.

*Numbers in the brackets are the references to the studies using FET-structure test STQ-77 on clinical samples. ↑–higher than optimal and ↓–lower than an optimal expression of the system and temperament traits*.

* The associations that were not proposed in the FET but were received in the studies.

Neurochemically-based consistent behavioral patterns in healthy people emerge as temperament traits (T-CBP) and even giftedness (G-CBP) (27) (not discussed here), and, in the case of strong neurochemical dysregulation—as symptoms of psychopathology in psychiatric cases, marked here as clinical CBP (C-CBP). Here we illustrate many symptoms of psychopathology can be presented as combinations of below- or above-optimal levels of performance of the systems described in this review. Space does not permit a detailed discussion but lets us briefly mention several examples.

The O system of homeostatic maintenance, especially the 5-HT system, is well-known as a factor of Major Depression (MD), and “signature” symptoms of MD are low physical endurance (fatigue), low social endurance (social withdrawal), loss of appetite and sleep problems (the last two problems are regulated by the endocrinal system, under hypothalamic NPs and 5-HT control). Another component of the O system is gut-brain cooperation, which includes the production of tryptophan (needed for 5-HT) and endorphins (suppressing HPA arousal and cytokines and so inducing relaxation and security). Lack of endorphins (binding to MORs) leads to inability “to like” whereas, as Berridge's group suggested, low DA might be associated with an inability “to want” (217) (i.e., inability to put generate a preferable program of actions). Since MOR influences the DA release, a combination of these two deficiencies might be subjectively experienced as a lack of motivation and interest. In cases of eating disorders or antibiotic treatment of infections that suppress guts microbiota, the production of local neurotransmitters is suppressed too, including endorphins (163–165, 218, 219). This can produce the aforementioned core symptoms of depression but also symptoms of anxiety disorders, due to lack of endorphins that usually suppress stress hormones (218, 219).

Cases of over-performance of this system are rarely discussed in the literature, but, as a clinical psychologist, I have seen many of them in my practice. For example, a patient with Ulcerative Colitis (i.e., with an overactive immune system affecting gut microbiota) reported having childhood and adolescence marked by frequent episodes of depression and anxiety. However, remarkably, in adulthood, they now report an almost opposite pattern (likely due to compensatory mechanisms in their bode and taking medications for a long time). This CBP includes low excitability about potential underachievement, no feeling of hopelessness or helplessness, severe problems with wanting, liking, planning and getting enthusiastic about anything, having remarkable stress resilience and full acceptance of who they are, in terms of social status. Chronic exposure to MOR stimulants affect 5-HT and BDNF systems causing a decrease in endurance (149, 193). This, however, doesn't make people anxious about their low physical capacities, especially if there are supportive SEPs to accommodated their non-demanding lifestyle.

The OO system of sustained attention, when underperforming, contributes to a low ability to concentrate on tasks, as a symptom of ADHD but also in thought disorders. Deficiencies in the 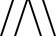 system of probabilistic processing are known as low intelligence and learning disabilities, or, on the other pole of dysbalance, as an excessive need for novel experience.

Much psychopathology can be linked to the OR-driven !I?-system of dispositional emotionality as the density of these receptors is a flexible system (41). Indeed, MOR upregulation (i.e., increased receptor density) has been linked to extreme dysphoria as seen in many cases of psychopathology, and irritability as seen in Major Depression (214), Post-traumatic Stress Disorder (220), Borderline Personality Disorder (BPD) (187, 221–224) and Attachment Disorders (188, 225). Sadness in BPD patients was also associated with a greater reduction of binding potential, in comparison to healthy patients (221), highlighting the idea of “broken mechanisms” in the restoration of chemical cycles, rather than a deficient amount of a specific NT, as a biomarker of pathology. We can hypothesize that in cases of MOR downregulation (low receptor density), people might experience a dispositional, often negligent satisfaction with “life as is,” low productivity, high agreeableness and dispositional positive mood. KOR upregulation of receptors might lead to a sense of “deflation,” lack of interest and low perceptual arousal, highlighted in the phenomenon described by George Koob as “hyperkatifeia” (207). The opposite pole of KOR dysregulation can be seen in the symptoms of behavioral agitation and enhanced perceptual sensitivity seen in Generalized Anxiety Disorder (197, 198, 200, 208–211), often comorbid with alcoholism affecting KORs. Extensive studies in addiction research give numerous examples the impact of disregulated OR systems, such as induced by the overuse of stimulants, on the speed of integration of behavior (i.e., plasticity, tempo and impulsivity).

In terms of the []-system of programming and the [[]]-system of sorting-out pre-fab units of action, deficiencies in behavioral plasticity (rigidity) are seen in the repetitive behavior and rituals of OCD patients (120–122, 145), when the [[]] system is overactive. Overactivity of DA-based “prioritizing” system in cognition is seen in schizophrenia (115–117) and psychoticism (117–119). The problems in ease of initiation and integration of behavior regulated by the [] and [[]] systems, emerge as a dramatic slowdown in actions, commonly seen in Major Depression (205) and Parkinson Disease (100, 143, 212). The 5-HT system, as part of homeostatic maintenance regulation, provides important support to many areas of the brain and so to all other aspects of behavior, including behavioral plasticity. This might explain links of 5-HT deficiency with impulsivity, however it appears that deficiencies in the DA system also can lead to impulsivity (142, 143), as a compromised construction of actions (213, 214). Finally, the deficiencies in cortical NE-ACh systems providing orientation and probabilistic processing are known as learning disabilities and low intelligence (69, 71, 73, 77, 78).

### A Psychiatric Disorder Rarely Presents as a Single Biomarker Problem

At home, when one functional system, such as a kitchen or bathroom goes out of order, we usually adjust and keep on going. However, when two or more functional systems are out of order, we start consider moving. Likewise, in psychopathology, if one neurochemical system is out of balance, other systems normally compensate for it. When two or more systems go out of balance, they amplify each other's dysfunction and affect many aspects of the integration of behavior. These changes, in turn, receive feedback from multiple levels of the environment, reinforcing them.

For example, an imbalance between the supply of endorphins and binding to MOR sites can generate a feeling of dysphoria and a low ability for “liking” (217); under-performance in DA networks in the ventral striatum can generate a deficiency in the programming of actions, low abilities for “wanting” (217) and starting new actions; a deficit of tryptophan (a component needed to produce serotonin) can generate sleep problems, a feeling of weakness, fatigue and low capacity. One of these cases by itself is common in healthy individuals and known as grumpiness, tiredness, insomnia, amotivation and rigidity. However, when all of them occur in one person, they amplify the feeling of low capacities, lack of interest and increase the severity in loss of functioning, resulting in Major Depression. Somatic illnesses are often comorbid with Major Depression, and we need to be more transparent about the underlying causes of this comorbidity. Many somatic illnesses affect the functioning of microbiota producing endorphins and tryptophan, and, if the illness involves infections, it also increases cytokines that challenge the functioning of glial cells, another component of the O-system (218). The association of the 5-HT system with the regulation of sleep, as a function of the activity of pineal gland depending on sunlight, can explain high rates of seasonal depression in Northern countries. During winter months, these countries have limited daylight time, and people, whose tryptophan production and supply to the brain were already challenged by other factors, can be more prone to seasonal depression.

As another example, the probabilistic processing system allows a person to capture the frequency, commonality and rarity of events, and derive “rules of engagement” with reality; it does not substitute for the system of sustained attention OO, even though when these systems work together, the result is a cognitively sharp individual. When one of these systems is compromised, it can be seen as average intelligence, poor concentration or insufficient attention to learning material, and, with effort and tools, a person can successfully compensate for it. However, when all of these systems are compromised, this can emerge as learning disabilities (inability to learn and comprehend new knowledge) and low intelligence.

All of this suggests that looking for specific single biomarkers for specific psychiatric diagnoses might not be very fruitful. However, if we go with a biomarkers-by-the-bunch approach, it is still useful to know what each member of the “bunch” has to contribute to behavioral regulation.

### Psychiatric Disorders Come With “Environmental Bubbles” Supporting Their CBP

Environmental reinforcement of Major Depression can be seen in families that support a lifestyle involving lack of physical exercise, bad diets, overuse of antibiotics, normalizing irritability, fatigability, dependency on other people's services, overuse of “orienting” activities such as watching TV and permitting poor sleep hygiene. It would be naïve to think that without this kind of support, people wouldn't experience Major Depression—they probably would. However, it is all matter of how long it takes for neurochemical cycles to come back to the functional level. Histories of wars and natural disasters showed that in those circumstances the rates of MD are low, despite major losses and objective reasons to be hopeless. At the same time, the US or Australia, which have not recently suffered from wars related to invasions on their territory with massive loses of property or population, have among the highest rates of MD. This suggests that the duration of MD might be malleable and might depend on the environmental infrastructure, for example supporting symptoms of hypochondria and somatization that could progress to MD if reinforced.

Similarly, civilized societies developed infrastructures to assist people with limited sustained attention and memory by providing them tools such as office supplies and computers. Environmental compensation for low probabilistic processing can be seen in the infrastructure of experts, counselors, media, science, giving advice to people and selecting information for them. Environmental compensation for a need for new sensations created not only the tourism industry but also the illicit drug use infrastructure and the overuse of legal psychostimulants, and includes the infrastructure of rehab centers, counselors and media attention to people with excessive sensation seeking. The same environmental bubbles can be seen involving people incapable of choosing their actions or performing manual work, or people with mood, anxiety or delusional disorders.

Thus, when we are working on psychiatric taxonomies and their biomarkers, it might be helpful to include a parameter for environmental settings noting features of where a person lives, as an additional marker indicating the severity of the illness. If patients describe their everyday life as a struggle to fit into the environment, paradoxically it can have more potential for treatment and less severity than when patients have established their learned helplessness patterns and the infrastructure that supports these patterns. Using 12-component FET framework could be used for the classification of context, where formal aspects of situations are assessed in terms of urgency, novelty, complexity, demands for endurance and dexterity (3).

### Arguments in Favor of a Unified Classification of Bio-Behavioral Diversity

There are many arguments in favor of a unified classification of bio-behavioral diversity that would include temperament of healthy people and cases of psychopathology (12, 26, 213). First, there is a commonality of neurochemical supports underlying these consistent behavioral patterns. Second, it is easier to study weak deviations in functional contributions of neurochemical systems to behavioral regulation using models of temperament on healthy subjects than to study substantial deviations in these same systems using clinical cases. After all, psychopathology very often comes with various comorbidities and ethical constraints, and this complicates the collection of samples and interpretation of results.

Third, psychiatric deviations from the norm don't develop overnight: typically, it takes time to establish clinically significant consistent behavioral patterns. Moreover, there is no sharp division between people with diagnosed psychopathology and people who are not diagnosed with it. There is a continuum between distinctly healthy and distinctly ill people (226), and most people could be put into the gray area between these two extremes, just having some “weak spots” in their neurochemical, behavioral regulation. They likely need to know how to deal with their weak spots without putting a psychiatric label on them.

It is essential, therefore, that our bio-behavioral taxonomies include all of the numerous shades of CBP between temperament and psychopathology.

## Summary

This Theme Issue is focused on principles of bio-social complexity of behavioral regulation, and when it comes to complexity, much could be said, especially in regards to the regulation of human behavior. Details of the neurochemical systems of behavioral regulation were provided in this review in order to illustrate of their different functionality but did not cover everything that should be said about them. As noted, there is a diversity of receptors within each of these systems, each having its own functional specificity.

The take-home message of this review was to suggest that, when developing of classifications of bio-behavioral individual differences, including psychiatric taxonomies, it is useful:

– to look at the benefits of a Functional Constructivism approach when analyzing “messy” matters of diverse and complex neurochemical systems; this approach highlights several universal functions in generative processes that could help classify neurochemical biomarkers and consistent behavioral patterns based on them;
– to keep in mind that behavior is being generated anew based on current, past and future considerations of the state of the body and context; this makes statistical and mathematical approaches relying on “counting and comparing heads” not very applicable”;
– to look for new formalisms of functional differentiation between systems of behavioral regulation; the FET model is an example of using such formalisms, as illustrated in Table 1 and Figure 3.
– to propose a concept of Specialized Extended Phenotype (SEP), as a “bubble”-infrastructure of social and physical nature that accommodate specific (dis)abilities; identification of psychiatric disorders, therefore, might benefit from reference to the presence or absence of congruent SEPs that could support healthy or dysfunctional CBPs;
– to consider a unification of taxonomies of bio-behavioral differences in healthy people (temperament traits) and in psychiatric cases: an example of a neurochemically-based model of such a taxonomy was briefly introduced in this paper and illustrated in Tables 2, 3.

Here we toyed with special symbolics, to facilitate the development of new “transient” mathematics that can handle the context-dependent and generative nature of the behavior. The main idea was to point to the correspondence of functional differentiation between neurochemical systems and formal functional aspects of behavior that could be used in future psychological modeling. The limitation of this review is that these are early attempts to map functionality of neurochemical biomarkers and CBP for the purposes of CBP classifications, and much more work should be done. Moreover, since any behavior, even mental and barely observable, relates to existing environmental infrastructure and so these classifications might benefit from the incorporating with classifications of professionals and functional SEPs using the same 12 categories (dynamical features of situations listed in Table 1).

## Author Contributions

The author confirms being the sole contributor of this work and has approved it for publication.

## Conflict of Interest

The author declares that the research was conducted in the absence of any commercial or financial relationships that could be construed as a potential conflict of interest.

## Publisher's Note

All claims expressed in this article are solely those of the authors and do not necessarily represent those of their affiliated organizations, or those of the publisher, the editors and the reviewers. Any product that may be evaluated in this article, or claim that may be made by its manufacturer, is not guaranteed or endorsed by the publisher.

## Abbreviations

FET: Functional Ensemble of Temperament
FC: Functional Constructivism
CBP: consistent behavioural patterns
N/C: balance between Needs and Capacities
NP: neuropeptides
Neurotransmitters: 5-HT: 5-hydroxytryptamine (serotonin)
ACh: acetylcholine
NE: noradrenaline
DA: dopamine
NP: neuropeptides
Glu: glutamate
GG: GABA and Glu
H: histamine
A: adenosine
ORE: rexins
SubP: Substance P
α1(2): alpha-1(2) adrenergic receptors
GPCR: -G protein–coupled receptors
KOR, MOR, DOR: kappa-, mu- and delta-opioid receptors correspondingly. Brain structures: (d, m, c)RN, (dorsal, median, caudal) raphe nucleus
(d, m)PFC -(dorsal, medial): prefrontal cortex
OFC: orbito-frontal cortex
HT: hypothalamus
HC: hippocampus
BF: basal forebrain
LC: locus coeruleus
(dL, V)TA, (dorsolateral, ventral): tegmental area
Th: thalamus
AM: amygdala
NAc: nucleus accumbens
Caud: caudate nucleus in ventral striatum
Put: putamen
STN: subthalamic nucleus
PPN: pedunculopontine nucleus
Crbm: cerebellum
GP(i, e): globus pallidium (internal, external)
SNc/r: substantia nigra pars compacta/ reticulata
(s, p)ANS: (sympathetic, parasympathetic) autonomous nervous system
HPA: hypothalamic-pituitary-adrenal axis
HPG: hypothalamic-pituitary-gonadal axis
HCA: hypothalamic-catecholamines axis.
